# Antifungal Susceptibilities of Rare Yeast Isolates

**DOI:** 10.3390/jof11090645

**Published:** 2025-09-01

**Authors:** Deniz Turan, Zafer Habip, Hakan Odabaşı, Esra Dömbekçi, Narin Gündoğuş, Merve Özmen, Sebahat Aksaray

**Affiliations:** 1 Istanbul Provincial Health Directorate ISLAB-2 Central Laboratory, Prof. Dr. Süleyman Yalçın City Hospital, Kadıköy, 34722 Istanbul, Türkiye; 2 Department of Medical Microbiology, Medeniyet University Faculty of Medicine, Kadıköy, 34720 Istanbul, Türkiye; drzaferhabip@gmail.com (Z.H.); drmerveozmen1@gmail.com (M.Ö.); 3 Department of Medical Microbiology, University of Health Sciences, Sancaktepe Sehit Ilhan Varank Training and Research Hospital, Sancaktepe, 34785 Istanbul, Türkiye; odabasi83@hotmail.com (H.O.); esra.dombekci@saglik.gov.tr (E.D.); naringun@gmail.com (N.G.); 4 Department of Medical Microbiology, University of Health Sciences, Hamidiye Medical School, Usküdar, 34668 Istanbul, Türkiye; aksarays@hotmail.com

**Keywords:** rare yeasts, identification, antifungal susceptibility testing, clinical breakpoints

## Abstract

The recent increase in the number of rare yeasts isolated from clinical specimens is a cause for concern, requiring accurate identification of these yeasts and assessment of their antifungal susceptibility to guide treatment. In this regard, we identified 196 rare yeasts isolated from various clinical specimens, mostly urine and respiratory tract specimens of patients hospitalized in intensive care unit and wards, using MALDI-TOF MS, and assessed their susceptibility to amphotericin B, fluconazole, voriconazole, posaconazole, itraconazole, isavuconazole and anidulafungin using the EUCAST broth microdilution method. Among the rare yeast species we isolated, *Candida lusitaniae* (13.8%) was the most common, followed by *Magnusiomyces capitatus* (13.3%), *Candida fabianii* (12.2%), and *Trichosporon asahii* (11.7%). Antifungal susceptibility testing revealed high echinocandin MIC values against *Magnusiomyces* spp., *Trichosporon* spp., and *Rhodotorula mucilaginosa* isolates. Similarly, we found high MIC values for fluconazole against the isolates of *Magnusiomyces* spp., *T. asahii*, *R. mucilaginosa*, and several *Candida* spp., including *Candida guilliermondii*, *Candida pararugosa*, *Candida rugosa*, *Candida pelliculosa*, *Candida norvegensis*, and *Candida fabianii*. We found similar MIC values across phylogenetically closely related species. In conclusion, prompt identification of rare yeasts and assessment of their antifungal susceptibilities are essential for effective treatment of the infections they cause.

## 1. Introduction

Rare yeasts that cause superficial infections in hosts with normal immune systems but can cause invasive fungal infections associated with high mortality and morbidity rates in immunosuppressed hosts or the presence of underlying risk factors are being isolated more commonly [1,2].

All phenotypic identification systems, matrix-assisted laser desorption ionization time-of-flight mass spectrometry (MALDI-TOF MS), and molecular approaches can contribute to the identification of rare yeasts [2]. However, traditional fungal identification methods based on examination of morphological and phenotypic characteristics are complicated by the diversity of organisms that can cause infections [3,4]. In MALDI-TOF MS systems, which are widely used in microbiology laboratories today, the difficulty of updating the nomenclature in the database prevents clinical microbiology laboratories from successfully adapting to new species names [4].

The widespread use of molecular approaches in fungal identification in recent years has led to radical changes in fungal nomenclature and taxonomy. Long-recognized species have been transposed to new genera based on genotypic comparisons, or new genera and species have been created that include new organisms identified by detailed phylogenetic analyses [3,4]. Recent changes to the Fungal Nomenclature Code have also led to dramatic changes in the nomenclature of medically important molds and yeasts [5]. The binomial nomenclature system, which stipulated giving separate names to anamorphic (asexual) and teleomorphic (sexual) forms of fungi, was abandoned as of 1 January 2013 [5,6,7].

To reduce the instability caused by unnecessary and temporary nomenclature in this process, working groups and committees established under the auspices of the International Commission on the Taxonomy of Fungi (ICTF) and the Nomenclature Committee for Fungi (NCF) have published lists of protected and rejected names of key species/genera for which definitive changes have been approved [3]. Information on the classical names most commonly used in clinical laboratories and the recommended names to be reported for clinical use can be accessed from the Atlas of Clinical Fungi, Index Fungorum, and MycoBank [8,9,10].

As with their nomenclature, antifungal susceptibility values for rare yeasts are also not well established. In fact, the European Committee on Antimicrobial Susceptibility Testing (EUCAST) has not reported clinical breakpoints for antifungal drugs against rare yeasts due to insufficient evidence, except for commonly isolated *Candida* species and *Cryptococcus neoformans* [11]. On the other hand, based on the study by Astvad et al., EUCAST developed a classification for the criteria for assessing antifungal drugs to be used against different fungal species based on two key assumptions [12]. The first assumption is that genetically related isolates would have similar pathogenicities and intrinsic susceptibility patterns. The second assumption is that rare yeasts, even those not phylogenetically related to other species, will likely respond to treatment, provided their minimum inhibitory concentration (MIC) values are similar to those observed in wild-type isolates of other, more commonly isolated species. Current treatment recommendations based on in vivo efficacy data and clinical experience were also taken into account in the EUCAST classification [11,13]. Accordingly, provisional MIC values were determined to guide amphotericin B, anidulafungin, fluconazole, and voriconazole treatments against thirty different yeasts until official clinical breakpoints are set by EUCAST [12].

In view of the foregoing, this study was carried out to identify rare yeasts isolated from various clinical specimens and assess their antifungal susceptibilities to amphotericin B, fluconazole, voriconazole, posaconazole, itraconazole, isavuconazole, and anidulafungin.

## 2. Materials and Methods

### 2.1. Fungal Isolates

The study material consisted of 196 rare yeast collection isolates sent to the ISLAB-2 Central Mycology Laboratory for identification and antifungal susceptibility testing from hospitals affiliated with the 2nd Service Region of the Istanbul Provincial Health Directorate between 1 June 2023 and 30 October 2024, and identified by matrix-assisted laser desorption ionization–time of flight mass spectrometry (MALDI-TOF MS) (VITEK MS and VITEK MS PRIME, bioMérieux, Marcy l’Etoile, France). Identification of the isolates was supported by their macroscopic and microscopic appearances on Sabouroud Dextrose agar [(SDA), (RTA, İstanbul, Turkey)] and Candida chromogenic agar (RTA, İstanbul, Turkey). The isolates were stored at −80 °C until testing, revived by passage twice in SDA prior to testing, and incubated at 36 ± 1 °C for 18–24 h.

### 2.2. Antifungal Susceptibility Testing

Antifungal susceptibility tests were carried out using the broth microdilution method per EUCAST E.Def 7.4 guidelines [14]. The antifungal agents tested were obtained from Sigma–Aldrich Chemical Co., St. Louis, MO, USA. The MIC values of amphotericin B, fluconazole, posaconazole, itraconazole, voriconazole, isavuconazole, and anidulafungin against the isolates were determined. Growth was assessed visually after 24-h incubation at 37 °C and spectrophotometrically at 450 nm using a Multiskan Go (ThermoFisherScientific, Waltham, MA, USA) microplate reader. The MIC value was defined as the lowest concentration that caused a 90% reduction in growth for amphotericin B and a 50% reduction for other drugs compared to growth not involving an antifungal agent [15].

### 2.3. Statistical Analysis

SPSS 30.0 (Statistical Product and Service Solutions for Windows, Version 30.0, IBM Corp., Armonk, NY, USA, 2024) software package was used in the statistical analyses of the collected data. The results of the statistical analyses were expressed using descriptive statistics, i.e., mean ± standard deviation values and median with minimum and maximum values in the case of continuous variables, and frequencies (n) and percentage (%) values in the case of categorical variables. In comparing the differences in variables between the groups, the Mann–Whitney U test was used for numerical variables, and the chi-squared test was used for categorical variables. Probability (*p*) statistics of <0.05 were deemed to indicate statistical significance.

## 3. Results

**Figure 1:**
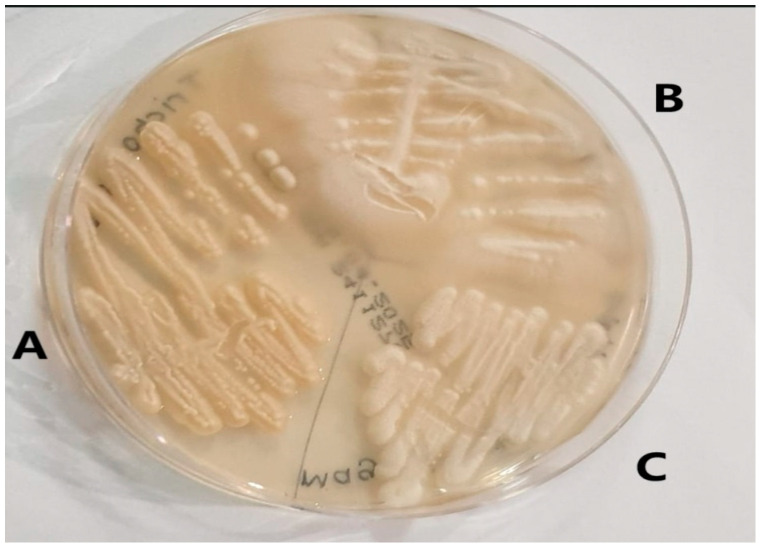
Images of rare yeasts on Sabouraud Dextrose agar. (**A**) *Trichosporon asahii.* (**B**) *Geotrichum capitatum.* (**C**) *Magsusiomyces capitatus* (From the study).

**Figure 2:**
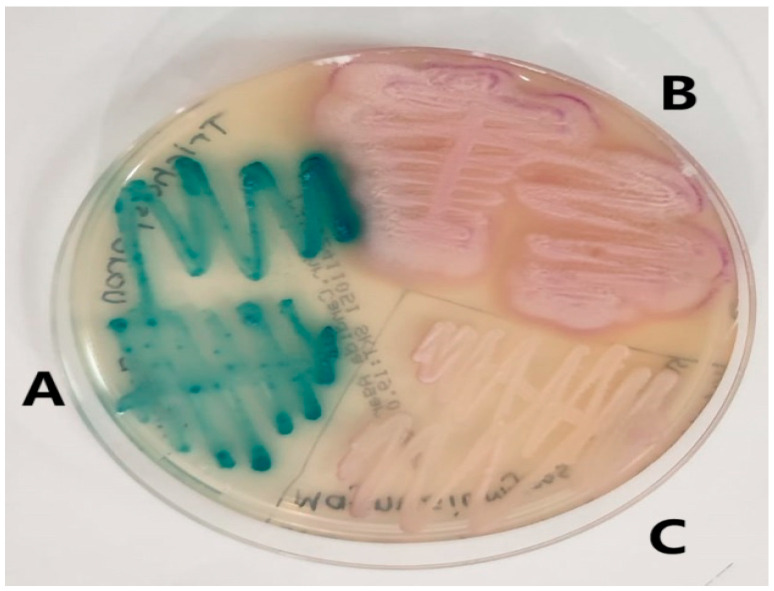
Images of rare yeasts on *Candida* chromogenic agar. (**A**) *Trichosporon asahii.* (**B**) *Geotrichum capitatum.* (**C**) *Magsusiomyces capitatus* (From the study).

We identified 196 rare yeasts and studied their antifungal drug susceptibility during the study period. Compared to other common *Candida* species, i.e., *Candida albicans* complex (1685), *Candida glabrata* complex (*Nakaseomyces glabratus*) (653), *Candida tropicalis* (441), *Candida auris* (*Candidozyma auris*) (417), *Candida parapsilosis* (307), *Candida kefyr* (*Kluvyeromyces marxianus*) (243), and *Candida krusei* (*Issatchenkia orientalis*) (76), rare yeasts accounted for 4.9% of all growth samples. The names and isolation rates of the 196 rare yeasts that make up the study material are shown in Table 1. The appearance of some yeasts isolated in the study on SDA is shown in Figure 1, and their appearance on *Candida* chromogenic agar is shown in Figure 2.

We isolated the rare yeasts mostly from the urine and respiratory tract specimens of patients hospitalized in the intensive care unit (ICU) and wards. No significant difference was found between the urine samples of patients hospitalized in the ICU and wards in terms of the isolation rates of rare yeasts compared to other common *Candida* species (*p* = 0.403). On the other hand, a significant difference was found between the respiratory tract samples of patients hospitalized in the ICU and wards in terms of the isolation rates of rare yeasts compared to other common *Candida* species (*p* < 0.001). The isolation rates of other *Candida* species were significantly higher than those of rare yeasts in the blood samples of patients hospitalized in the ICU and wards (*p* = 0.004). Among the rare yeast species we isolated, *Candida lusitaniae* (*Clavispora lusitaniae*) (13.8%) was the most common, followed by *Saprochaete capitata* (*Magnusiomyces capitatus*) (13.3%), *Candida fabianii* (*Cyberlindnera fabianii*). (12.2%), and *Trichosporon asahii* (11.7%). Demographic characteristics of patients from whom rare yeasts were isolated and isolation rates by sample type are shown in Table 2.

EUCAST has not provided clinical breakpoints for antifungal drugs against rare yeasts. Therefore, it is recommended that evaluations of clinical breakpoints for antifungal medicines against rare yeasts should be made according to clinical breakpoints specified for either species to which rare yeasts are phylogenetically similar or wild isolates of other common species. We found high MIC values against *Magnusiomyces* spp., *Trichosporon* spp., and *Rhodotorula mucilaginosa*, which reportedly have intrinsic echinocandin resistance. Similarly, we found high MIC values for fluconazole against the isolates of *M. capitatus*, *Saprochaete clavata* (*Magnusiomyces clavatus*), *T. asahii*, and *R. mucilaginosa*, as well as *Candida guilliermondii* (*Meyerozyma guilliermondii*), *Candida pararugosa* (*Diutina pararugosa*), *Candida rugosa* (*Diutina rugosa*), *Candida pelliculosa* (*Wickerhamomyces anomalus*), *Candida norvegensis* (*Pichia norvegensis*), and *C. fabianii*, which were formerly included in *Candida* species. Antifungal susceptibilities of yeasts isolated from more than one sample are shown in Table 3, whereas antifungal susceptibilities of rare yeasts grown in only one sample are shown in Table 4.

## 4. Discussion

The accurate identification of rare yeasts and determination of their antifungal susceptibilities are crucial for guiding treatment and vaccination. Some antifungals have limited spectra of activity in rare yeast species due to intrinsic resistance. As with fluconazole, the antifungal efficacies of other antifungal drugs also vary [16]. Among the 196 rare yeast species we isolated, *C. lusitaniae* (13.8%) was the most common, followed by *M. capitatus* (13.3%), *C. fabianii* (12.2%), and *T. asahii* (11.7%). We found high MIC values of anidulafungin against isolates of *Magnusiomyces* spp., *Trichosporon* spp., and *R. mucilaginosa*, and of fluconazole against isolates of *M. capitatus*, *M. clavatus*, *T. asahii*, and *R. mucilaginosa*, as well as isolates of other species such as *M. guilliermondii*, *D. pararugosa*, *D. rugosa*, *W. anomalus*, *P. norvegensis*, and *C. fabianii*.

EUCAST has not reported clinical breakpoints for antifungal drugs against rare yeasts due to insufficient evidence. Then again, it has been recommended that antifungal drugs used in the treatment of isolates to which rare yeasts are genetically related be also used in the treatment of rare yeast infections, based on the assumption that their pathogenicity and intrinsic susceptibility patterns would be similar, as well as antifungal drugs that exhibit MIC values against wild-type isolates of other frequently isolated species that are not phylogenetically related to rare yeasts similar to those against isolates genetically related to rare yeasts [12,16].

In a study on rare yeasts isolated from fungemia cases, Tepe et al. [17] found that *Trichosporon* spp., *Saprochaete* spp., *Cryptococcus* spp., and *Rhodotorula* spp. had intrinsic resistance to echinocandin. We also isolated yeasts with intrinsic echinocandin resistance, such as *Trichosporon* spp. and *Saprochaete* spp., although rare (7.7%), among the rare yeasts we isolated from fungemia cases. Therefore, in patients with predisposing risk factors for the development of fungal infections, rare yeasts as well as *Candida* species should be kept in mind when deciding on empirical treatment. In a study on antifungal susceptibilities of rare yeasts according to EUCAST standards, Stavru et al. categorized the MIC values of antifungal drugs against rare yeasts for which there are no epidemiological breakpoints as susceptible/reduced susceptible in comparison with the epidemiological breakpoints of *C. albicans*. Accordingly, they reported that species of *Meyerozyma*, *Diutina*/*Kodamaea*, and *Pichia* clades showed reduced susceptibility to azoles, and *Candida haemulonii*, *Candida pseudohaemulonii*, and *Candida duobushaemulonii* species, which are closely related to *C. auris*, also showed reduced susceptibility to some antifungal drugs, mainly azoles [16]. In parallel, high MIC values were reported for azoles against *Candida inconspicua*, *C. pararugosa*, and *P. norvegensis* isolates [18]. We also found high MIC values of fluconazole against *M. guilliermondii*, *P. norvegensis*, *D. pararugosa*, *D. rugosa*, *Candida palmophilia*, *C. haemuli*, *M. capitatus*, *T. asahii*, and *R. mucilaginosa*.

Echinocandins are recommended as first-line treatment for invasive candidiasis [19]. In a study investigating echinocandin resistance in yeasts using the broth microdilution method based on a MIC threshold value of ≥0.5 mg/L, Ollivier et al. reported that echinocandins had low MIC values against isolates of *C. lusitaniae*, *Candida dubliniensis*, *M. guilliermondii*, *P. inconspicua*, *Saccharomyces cerevisiae*, *C. haemuli*, and *W. anomalus*, and high MIC values against isolates of *M.capitatus*, *M. clavatus*, *T. asahii*, *R. mucilaginosa*, and *Yarrowia lypolitica*. We found MIC values generally similar to those reported in the above studies, except against isolates of some specific genera and species, which acquired resistance due to previous exposure to antifungal drugs [19].

In a study investigating the antifungal susceptibilities of rare yeasts isolated from various clinical samples, it was reported that amphotericin B, fluconazole and itraconazole showed high MIC values against *C. haemuli* isolates, fluconazole and caspofungin against *M. capitatus* isolates, fluconazole against *K. ohmeri* isolates, and fluconazole, posaconazole and caspofungin against *R. mucilaginosa* isolates [20]. We also found similar MIC values against these yeasts.

Onychomycosis is another fungal infection in which rare yeasts have been isolated as causative agents. A study reported *Candida orthopsilosis* (n = 9) as the most commonly isolated rare yeasts causing onychomycosis, followed by *C. lusitaniae* (n = 2), *W. pararugosa* (n = 2), *Naganishia diffluens* (n = 2), *W. anomalus* (n = 2), *C.fabianii* (n = 1), and *Meyerozyma caribbica* (n = 1) [21]. In the same study, fluconazole was found to have high MIC values against most of these species. Of the rare yeast isolates we identified, 5.1% were causative agents of onychomycosis. Of these isolates, *R. mucilaginosa* and *T. asahii* were isolated in three cases each, *C. fermentati* and *M. guilliermondii* in two cases each, and *C. orthopsilosis* and *Zygosaccharomyces rouxii* in one case each. Fluconazole exhibited high MIC values against all these species.

This study has some limitations. First, the epidemiology of rare yeasts varies across regions, countries, and even institutions. Although the participating centers had a large patient population and diverse populations, this study only presents data on strains identified in the ISLAB-2 service area. Therefore, our findings may not reflect species distribution or resistance patterns in other regions with different climates or patient demographics. Second is the lack of clinical data. The study included only in vitro antifungal susceptibility results. Clinical characteristics such as patient risk factors and thirty-day mortality rates could not be examined in detail due to limited access to clinical data. This situation caused insufficient confirmation of the clinical validity of the obtained MIC values. Another limitation is that molecular methods cannot be used in the identification of rare yeasts or in the search for antifungal resistance genes. In our study, yeasts isolated from axenic cultures, which were thought to have preserved their morphological, physiological and genetic characteristics, were identified by MALDI-TOF MS and supported by conventional methods. It has been concluded that identification should be supported by additional conventional methods to avoid possible misidentifications, especially in the identification of phylogenetically similar species. Finally, in this study, susceptibility results for seven traditional antifungal drugs were investigated. This presents a limited antifungal panel. In particular, in rare yeasts where antifungal resistance is observed, the inclusion of new antifungal drugs with different mechanisms of action, such as ibrexafungerp, rezafungin, fosmanogepix or oteseconazole, will allow the expansion of treatment alternatives and provide information for resistance monitoring programs.

## 5. Conclusions

EUCAST guidelines currently do not provide established clinical breakpoints for antifungal drugs against rare yeasts, yet recommend the use of antifungal drugs that exhibit MIC values against these rare yeasts similar to those against more common and possibly more pathogenic *Candida* species in the treatment of invasive infections in which rare yeasts have been isolated as causative agents. In this context, the identification and antifungal susceptibility results we obtained may contribute to epidemiological studies and guidelines on antifungal management of rare yeasts.

## Figures and Tables

**Table 1:** Rare yeasts isolated in the study.

Classical Name Most Commonly Used in Clinical Laboratories	Recommended Name to Be Reported for Clinical Use	n (%)
*Candida dubliniensis*	*Candida dubliniensis* ^ a,b,c^	18 (9.2)
*Candida fermentati*	*Candida fermentati* ^ a,b,c^	1 (0.5)
*Candida guilliermondii*	*Meyerozyma guilliermondii* ^ a,b,c^	5 (2.6)
*Candida haemulonis*	*Candida haemuli*^ a,b^ or *Candidozyma haemuli* ^c^	1 (0.5)
*Candida inconspicua*	*Candida inconspicua*^ a^ or *Pichia inconspicua* ^b,c^	21 (10.7)
*Candida lambica*	*Pichia fermentans* ^ a,b,c^	1 (0.5)
*Candida lusitaniae*	*Clavispora lusitaniae* ^ a,b,c^	27 (13.8)
*Candida lypolitica*	*Yarrowia lypolitica* ^ a^	2 (1)
*Candida metapsilosis*	*Candida metapsilosis* ^a,b,c^	3 (1.5)
*Candida norvegensis*	*Pichia norvegensis* ^ a,b,c^	2 (1)
*Candida orthopsilosis*	*Candida orthopsilosis* ^ a,b,c^	8 (4.1)
*Candida palmioleophila*	*Candida palmioleophila* ^ b,c^	1 (0.5)
*Candida pararugosa*	*Wickerhamiella pararugosa* ^ b,c^	3 (1.5)
*Candida pelliculosa*	*Hansenula anomala*^ a^ or *Wickerhamomyces anomalus* ^b,c^	2 (1)
*Candida rugosa*	*Diutina rugosa* ^ a,b,c^	3 (1.5)
*Candida fabianii*	*Cyberlindnera fabianii* ^ a,b,c^	24 (12.2)
*Cryptococcus neoformans*	*Cryptococcus neoformans* ^ a,b,c^	1 (0.5)
*Candida guilliermondii* var. *membranifaciens*	*Kodemea ohmeri* ^ b,c^	1 (0.5)
*Lodderomyces elongisporus*	*Lodderomyces elongisporus* ^ a,b,c^	1 (0.5)
*Rhodotorula mucilaginosa*	*Rhodotorula mucilaginosa* ^ a,b,c^	6 (3.1)
*Saccharomyces cerevisiae*	*Saccharomyces cerevisiae* ^a,b,c^	4 (2)
*Saprochaete capitata*	*Saprochaete capitata*^ b^ or *Magnusiomyces capitatus* ^c^	26 (13.3)
*Saprochaete clavata*	*Magnusiomyces clavatus* ^ b,c^	9 (4.6)
*Trichosporon asahii*	*Trichosporon asahii* ^ a,b,c^	23 (11.7)
*Trichosporon domesticum*	*Apiotrichum domesticum* ^ b,c^	2 (1)
*Zygosaccharomyces rouxii*	*Zygosaccharomyces rouxii* ^ b,c^	1 (0.5)

^a^ Atlas of Clinical Fungi; ^b^ Index Fungorum; ^c^ MycoBank.

**Table 2:** Demographic Distribution of Rare Yeasts.

Parameters	Rare Yeasts (n = 196)
Gender	
Male	109 (55.6)
Woman	87 (44.4)
Age	
mean ± SS	63.1 ± 22.9
Median (Min–Max)	70.5 (0–95)
Department	
Other	15 (7.7)
Service	96 (49)
ICU	85 (43.4)
Sample	
BAL	14 (7.1)
Sputum	26 (13.3)
Skin scraping	2 (1)
Tissue	3 (1.5)
Urine	94 (48)
Blood	15 (7.7)
Catheter	7 (3.6)
Peritoneal fluid	1 (0.5)
Nail	10 (5.1)
Tracheal aspirate	20 (10.2)
Vaginal swab	3 (1.5)
Wound	1 (0.5)

ICU: Intensive Care Unit; BAL: Bronchoalveolar Lavage.

**Table 3:** Distribution of Antifungal Susceptibilities of Rare Yeasts Growing in Multiple Samples.

Yeast (n)	AB		ANI		POSA		VOR		FLZ		ISV		ITRA	
	MIC Range	MIC 90	MIC Range	MIC 90	MIC Range	MIC 90	MIC Range	MIC 90	MIC Range	MIC 90	MIC Range	MIC 90	MIC Range	MIC 90
*Candida lusitaniae* (*Clavispora lusitaniae*) (27)	1–2	2	0.06–0.25	0.25	0.06–0.25	0.125	0.015–0.125	0.03	0.125–8	2	0.008–0.5	0.03	0.03–0.25	0.125
*Saprochaete capitata* (*Magnusiomyces capitatus*) (26)	1–2	2	2–>4	>4	0.5–2	1	0.5–2	2	2 –32	16	0.25–1	1	0.06–2	1
*Candida fabianii* (*Cyberlindnera fabianii*) (24)	1–2	2	0.015–0.25	0.125	0.5–1	0.5	0.125–1	0.25	4–32	16	0.06–0.25	0.125	0.125–1	0.25
*Trichosporon asahii* (23)	2–4	4	>4	>4	0.25–2	1	0.125–1	0.5	1– 32	8	0.125–0.5	0.5	0.5–1	1
*Candida inconspicua* (21)	1–4	2	0.015–0.125	0.03	0.25–0.5	0.5	0.25–1	0.5	16–>32	>32	0.015–0.5	0.25	0.06–0.5	0.25
*Candida dubliniensis* (18)	0.5–1	1	0.008–0.03	0.03	0.03–0.25	0.125	0.015–0.06	0.03	0.125–1	0.5	0.008–0.03	0.015	0.03–0.25	0.125
*Saprochaete clavata* (*Magnusiomyces clavatus*) (9)	1–4	2	2–>4	>4	0.5–2	2	0.5–1	1	8–16	16	0.5–1	0.5	0.25–1	1
*Candida orthopsilosis* (8)	1–2	1	0.5–2	2	0.06–0.25	0.25	0.125–0.25	0.125	1– 2	2	0.03–0.125	0.125	0.06–0.12	0.125
*Rhodotorula mucilaginosa* (6)	1–2	2	0.125–>4	>4	0.5–2	2	1– 8	8	2– 16	16	0.06–1	1	0.5–4	4
*Candida guilliermondii* (*Meyerozyma guilliermondii*) (5)	1	1	1–2	1	0.5–2	1	1– 4	2	8–32	16	0.5–>4	4	0.06–>16	16
*Saccharomyces cerevisiae* (4)	1	1	0.06–0.125	0.125	1	1	0.5–1	1	8–16	16	0.125–0.25	0.25	0.5–2	2
*Candida metapsilosis* (3)	1–2	1	0.25–0.5	0.5	0.125–0.25	0.125	0.06–0.125	0.125	0.5–2	2	0.03–0.125	0.03	0.06–0.125	0.06
*Candida pararugosa* (*Diutina pararugosa*) (3)	1	1	0.125–0.25	0.25	0.25–0.5	0.5	0.25–2	2	2– 8	8	0.125	0.125	0.25–0.5	0.5
*Candida rugosa* (*Diutina rugosa*) (3)	1–2	2	0.12–0.5	0.5	0.06–0.25	0.25	0.25–2	2	8	8	0.125	0.125	0.25–1	1
*Trichosporon domesticum* (2)	1–2	2	>4	>4	0.25–0.5	0.5	0.125–0.25	0.25	2	2	0.125	0.125	0.25–0.5	0.5
*Candida pelliculosa* (*Wickerhamomyces anomalus*) (2)	0.25–1	1	0.015	0.015	0.5–1	1	0.5	0.5	2–4	4	0.125–0.25	0.25	0.125–0.25	0.25
*Candida norvegensis* (*Pichia norvegensis*) (2)	2	2	0.12–4	4	0.25	0.25	0.06–4	4	8	8	0.25	0.25	0.25	0.25
*Candida lypolitica* (*Yarrowia lypolitica*) (2)	1	1	>4	>4	1–2	2	0.25	0.25	4–8	8	0.125–0.25	0.25	0.5	0.5

AB: Amfoterisin B; ANI: Anidulafungin; POSA: Posaconazole; VOR: Voriconazole; FLZ: Fluconazole; ISV: Isavuconazole; ITRA: Itraconazole; MIC: Minimal inhibitory concentration.

**Table 4:** Distribution of Antifungal Susceptibilities of Rare Yeasts Growing in a Single Sample.

Yeast	AB MIC	ANI MIC	POSA MIC	VOR MIC	FLZ MIC	ISV MIC	ITRA MIC
*Candida fermentati*	1	1	1	1	4	1	1
*Candida haemulonis* (*Candida haemuli*)	4	0.25	1	0.25	32	0.125	16
*Candida lambica* (*Pichia fermentans*)	0.25	0.015	0.5	0.5	4	0.25	0.25
*Candida palmophilia*	1	0.06	0.5	4	>32	1	1
*Cryptococcus neoformans*	2	>4	0.5	0.125	2	0.03	0.5
*Kodemea ohmeri*	0.25	4	0.25	0.125	8	0.125	0.25
*Lodderomyces elongisporus*	0.5	0.03	0.5	0.125	0.5	0.03	0.06
*Zygosaccharomyces rouxii*	1	0.015	0.03	0.03	0.125	0.06	0.25

AB: Amfoterisin B; ANI: Anidulafungin; POSA: Posaconazole; VOR: Voriconazole; FLZ: Fluconazole; ISV: Isavuconazole; ITRA: Itraconazole; MIC: Minimal inhibitory concentration.

## Data Availability

The original contributions presented in this study are included in the article. Further inquiries can be directed to the corresponding author.
